# Protective responses of tolerant and sensitive wheat seedlings to systemic and local zearalenone application – Electron paramagnetic resonance studies

**DOI:** 10.1186/s12870-021-03177-1

**Published:** 2021-08-21

**Authors:** Sieprawska Apolonia, Łabanowska Maria, Kurdziel Magdalena, Filek Maria, Skórka Magdalena, Barbasz Anna

**Affiliations:** 1 Institute of Biology, Pedagogical University, ul. Podchorążych 2, 30-084 Kraków, Poland; 2 Faculty of Chemistry, Jagiellonian University, ul. Gronostajowa 2, 30-387 Kraków, Poland

**Keywords:** Zearalenone, EPR, Wheat seedlings, Infection, Redox homeostasis

## Abstract

**Background:**

Mycotoxins are among the environmental stressors whose oxidative action is currently widely studied. The aim of this paper was to investigate the response of seedling leaves to zearalenone (ZEA) applied to the leaves (directly) and to the grains (indirectly) in tolerant and sensitive wheat cultivars.

**Results:**

Biochemical analyses of antioxidant activity were performed for chloroplasts and showed a similar decrease in this activity irrespective of plant sensitivity and the way of ZEA application. On the other hand, higher amounts of superoxide radical (microscopic observations) were generated in the leaves of plants grown from the grains incubated in ZEA solution and in the sensitive cultivar. Electron paramagnetic resonance (EPR) studies showed that upon ZEA treatment greater numbers of Mn - aqua complexes were formed in the leaves of the tolerant wheat cultivar than in those of the sensitive one, whereas the degradation of Fe-protein complexes occurred independently of the cultivar sensitivity.

**Conclusion:**

The changes in the quantity of stable, organic radicals formed by stabilizing reactive oxygen species on biochemical macromolecules, indicated greater potential for their generation in leaf tissues subjected to foliar ZEA treatment. This suggested an important role of these radical species in protective mechanisms mainly against direct toxin action. The way the defense mechanisms were activated depended on the method of the toxin application.

## Background

Maintaining redox homeostasis in plant cells under environmental stress is vital for proper plant development and yielding. The homeostasis disturbances result from the generation of excessive reactive oxygen species (ROS) surpassing the cell reduction capability [1]. Inactivation of the surplus ROS occurs through the activation/synthesis of enzymatic and non-enzymatic antioxidants, whose effectiveness depends, among others, on plant tolerance/sensitivity to stress [2]. Therefore, recognition of differences in the mechanisms of stimulating the stress resistance between cultivars belonging to the same species leads to obtaining new genotypes more tolerant toward the changes of the environmental conditions. As chloroplasts are the organelles in which ROS production may be one of the steps of photosynthesis, and as the correct course of photosynthesis is necessary for proper functioning of plants, the changes occurring in these organelles under stress are of particular importance.

The disturbance of direct electron transfer from reduced ferredoxin to O_2_ evokes changes in the oxidation state of paramagnetic metal ions (mainly Mn, Fe, Cu) that constitute the redox centers in proteins involved in the electron transfer in the photosystems [3]. The study of photosynthesis, with its redox reactions including changes in the oxidation state of metal ions or formation of radical species are carried out by different methods. One of them is electron paramagnetic resonance spectroscopy (EPR), which allows for recognition of paramagnetic centers, including Fe (III), Mn (II), Cu (II) and various kinds of radicals. High amounts of Fe (III) as well as Fe (II) ions are contained in ferritin, a protein whose basic function is iron storage. Under Fe deficiency, ferritin can serve as Fe donor, and under Fe excess as its accumulator [4]. This protective property of ferritin is particularly important, as high concentrations of iron ions can evoke the oxidative stress and lead to oxidative damage of proteins and membrane lipids. In our earlier studies Fe (III) species, the form of Fe ions determined by EPR method in standard conditions, were analyzed based on differences in the character of their signals registered at different temperatures. Variations in the content and character of manganese ions, occurring upon oxidative stress, were also studied, based on EPR spectra [5]. Characteristic hyperfine structure of manganese signals visible in the spectra of inorganic compounds of Mn (II) in oxide environments, for example in [Mn(H_2_O)_6_]^2+^ aqua-complexes allowed for differentiating these species from organic structures containing interacting manganese ions [6, 7]. Moreover, signal intensities of these manganese aqua-complexes were different in the spectra of each genotype, which allowed us to distinguish them from one another [5].

In addition to signals derived from paramagnetic metal ions, the EPR technique allows for the registration of radical spectra. The measurement of reactive radical species requires the use of a trapping technique, but stable organic radicals, so-called “long-lived radicals” could be recorded without much difficulty. Examination of these species is particularly important in the study of the mechanisms of oxidative stress, as it was shown that they are formed on organic molecules, mainly polysaccharides and proteins, as a result of “trapping” electrons generated in excess under stress [8].

One of the more prevalent fusaria toxins is zearalenone [9]. Their accumulation in plant cells initiates oxidative stress, which disturbs redox homeostasis and therefore is responsible for quantitative and qualitative crop loss. Additionally, mycotoxins taken with plant foods stimulate disease reactions in human and animal cells. In our earlier studies we found that zearalenone (ZEA) may be localized in the chloroplasts and may interact with these organelles [10, 11]. The accumulation of ZEA in the chloroplasts reduced the total content of chlorophyll in wheat cells, especially in the sensitive cultivar, indicating semi-destruction of the photosystem [12]. In this study, the observed effects on leaf chloroplasts resulted from a long-distance translocation of this toxin from the infected seeds. Another, equally possible way of infection by ZEA is foliar absorption, after colonization by *Fusarium*, leading to significant crop loss of agronomically important plants. Kornaś et al. [13] reported modifications in the chloroplast structure in cereal leaves after direct treatment with ZEA. These changes could be a consequence of ZEA mediated damage to chloroplast membranes resulting from ROS interactions with lipids and/or changes in the membrane hydrophilic/hydrophobic structure associated with location of the toxin [14]. The specific organization of chloroplast membranes, containing significant amounts of galactolipid fractions (MGDG and DGDG), can provide an environment in which ROS and free electrons may be trapped at sugar groups of these lipids creating long-lived radicals [5].

The aim of the presented research was to find out whether the way of ZEA application determined the oxidative changes in the chloroplasts and to what extent these potential changes depended on the resistance of plants to stress conditions. The oxidative potential was examined by biochemical methods in the isolated chloroplasts and verified using EPR technique to study the changes in the paramagnetic centers in the leaves of the tolerant and sensitive wheat cultivars subjected to intra- and extracellular ZEA treatment. EPR studies were performed directly on the leaves to prevent the influence of any additional stressors during sample preparation that could obscure the mechanism of ZEA action. Wheat cultivars were chosen for the research, as wheat is the most popular cereal in Europe, and the mechanism of its protection against mycotoxins is still not fully recognized.

## Results

### Biochemical analyses

The studies of the antioxidant enzymes in the chloroplasts obtained from the plants grown from the seeds soaked in ZEA as well as those in which the second leaf was brushed, revealed similar enzymatic activity (SODs, CAT and POX) in the control plants, depending on the tested genotype (Table 1). Generally, in cv. ‘Parabola’ all enzymes exhibited higher activity than in cv. ‘Raweta’. When the seeds were treated with ZEA, a reduced activity of all enzymes (as compared with control) was noted in both studied cultivars, however, the changes were more drastic in cv. ‘Raweta’. In the plants undergoing foliar ZEA treatment, the activity of all enzymes decreased in cv. ‘Raweta’, while cv. ‘Parabola’ demonstrated a boost in SODs and CAT activity.

**Table 1:** Activities of antioxidative enzymes (SOD: superoxide dismutase; CAT: catalase; and POX: peroxidases) in the chloroplasts of wheat cvs. ‘Parabola’ and ‘Raweta’ obtained from control plants, ZEA treated grains and ZEA treated leaves

	Parabola			Raweta		
	SOD	CAT	POX	SOD	CAT	POX
*ZEA treated grains*						
Kontrola	0.087 ± 0.004^a^	3.104 ± 0*.*006^a^	0.0321 ± 0*.*0005^a^	0.079 ± 0.005^a^	2.922 ± 0*.*003^a^	0.0311 ± 0*.*0006^a^
ZEA	0.065 ± 0.003^b^	3.079 ± 0*.*005^b^	0.0263 ± 0*.*0007^b^	0.040 ± 0.005^b^	1.606 ± 0*.*006^b^	0.0188 ± 0*.*0005^b^
*ZEA treated leaves*						
Kontrola	0*.*090 ± 0*.*004^a^	3*.*102 ± 0*.*005^b^	0*.*0351 ± 0*.*0006^a^	0*.*076 ± 0*.*002^a^	2*.*940 ± 0*.*004^a^	0*.*0308 ± 0*.*0006^a^
ZEA	0*.*109 ± 0*.*005^a^	3*.*502 ± 0*.*004^a^	0*.*0243 ± 0*.*0005^b^	0*.*049 ± 0*.*003^b^	1*.*642 ± 0*.*003^b^	0*.*0156 ± 0*.*0005^b^

Data represent the mean from three independent experiments (eight repetition for each independent experiment) ± standard error (SE). Different letters indicate significant differences between treatments; *P* ≤ 0.05

**Fig. 1:**
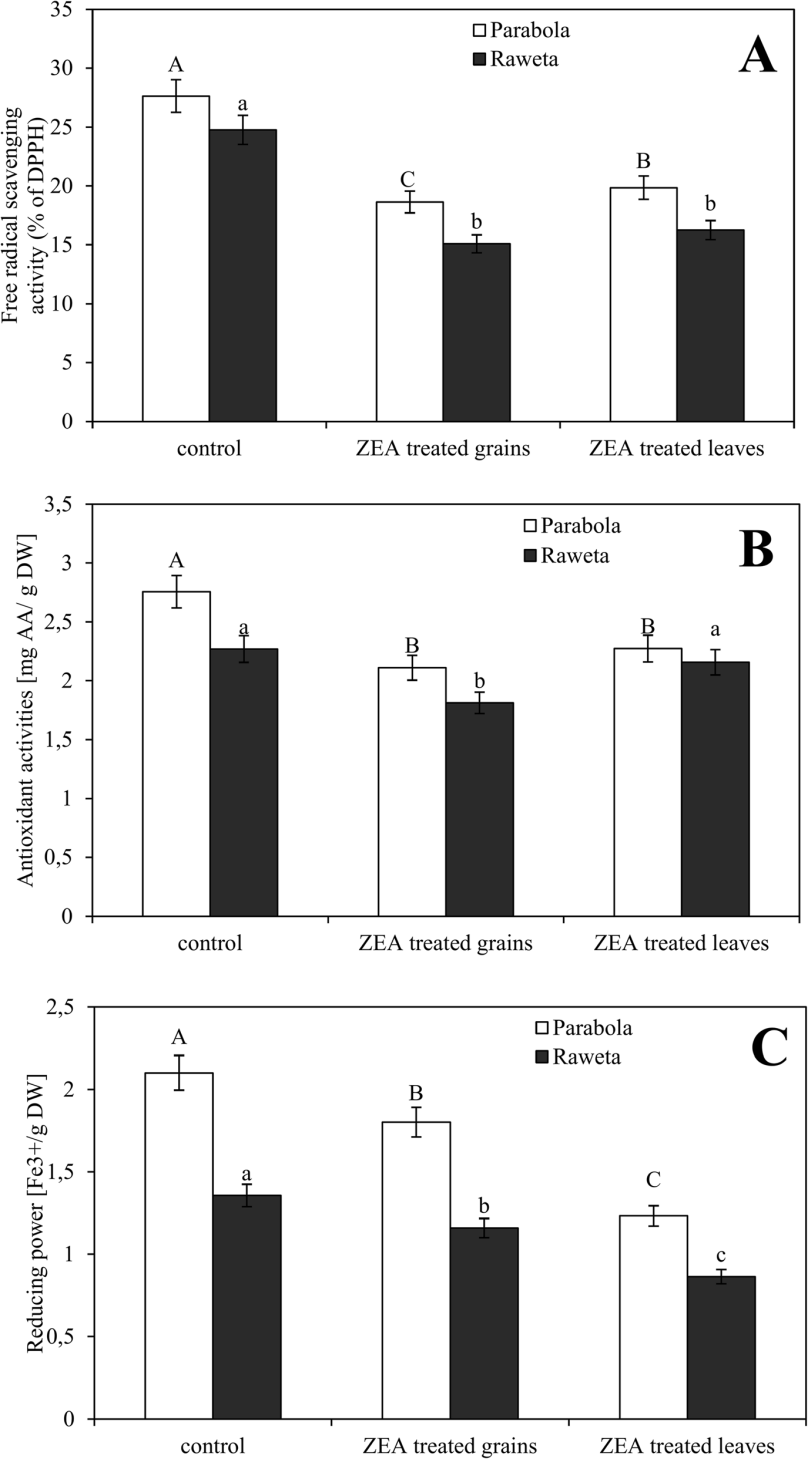
Antioxidant activity (expressed as percentage of reduced DPPH, the amount of ascorbic acid, AA and Fe^3+^ ions) in the chloroplasts of wheat cvs. ‘Parabola’ and ‘Raweta’ obtained from control plants, ZEA treated grains and ZEA treated leaves. Data represent a mean from three independent experiments ± standard error (SE). Different letters indicate significant inter-group differences, *p* ≤ 0.05

Antioxidant properties of the chloroplasts presented as the percentage of DPPH radical scavenging activity indicated that under control conditions the antioxidant activity was higher in cv. ‘Parabola’ chloroplasts than in cv. ‘Raweta’ ones (Fig. 1A). Both methods of ZEA application reduced the value of this parameter, however, it was slightly more visible in the chloroplasts obtained from plants grown from the grains infected by ZEA than in the chloroplasts isolated directly from the infected leaves. The changes were more noticeable in cv. ‘Raweta’.

Antioxidant activity, expressed as ascorbic acid concentration, was greater in the control chloroplasts of cv. ‘Parabola’ than those of cv. ‘Raweta’ (Fig. 1B). After ZEA treatment, the chloroplasts of cv. ‘Parabola’ exhibited a similar decrease in their antioxidant activity, irrespective of the application method. For cv. ‘Raweta’, lower values were obtained when grains were infected by the mycotoxin than when it was absorbed directly by the leaves.

In the calculations of the reducing power based on the system capability to deactivate electrons (Fe (III)/Fe (II) changes), the chloroplasts of cv. ‘Parabola’ were characterized by higher content of Fe (III) in control conditions than those of cv. ‘Raweta’. This meant that Parabola cells contained fewer free electrons (more efficiently neutralized by antioxidant systems) capable of reducing Fe (III) ions. ZEA application generally lowered Fe (III) levels, more significantly in the chloroplasts of plants directly treated with this mycotoxin (Fig. 1C).

### Visualization of ROS

**Fig. 2:**
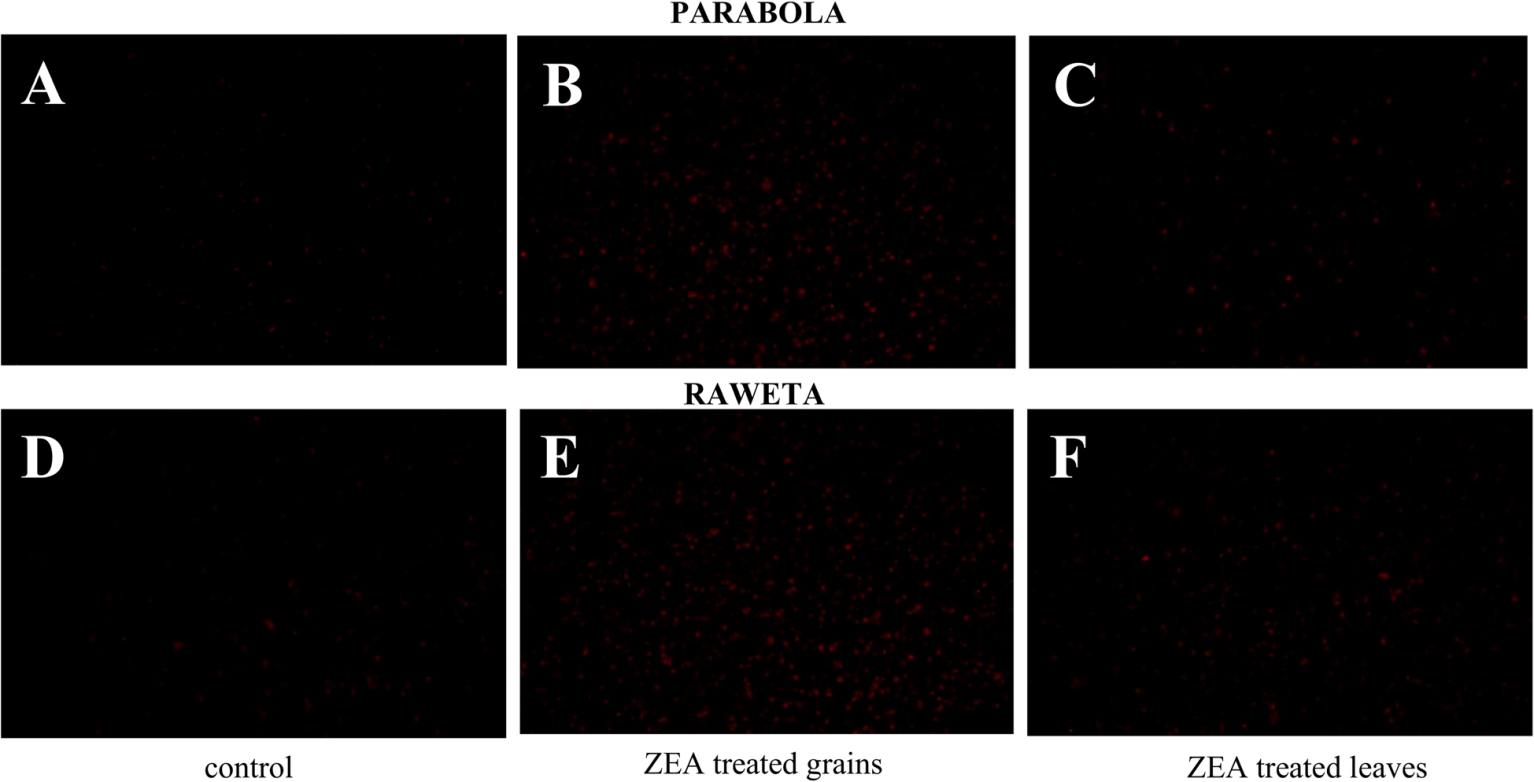
Superoxide radical activity in the chloroplasts of wheat cvs. ‘Parabola’ and ‘Raweta’ obtained from control plants, ZEA treated grains and ZEA treated leaves. Data represent a mean from three independent experiments ± standard error (SE). Different letters indicate significant inter-group differences, *p* ≤ 0.05

Microscopic observations revealed the presence of superoxide radicals in the tested chloroplasts, better visible in control samples of cv. ‘Parabola’ than those of cv. ‘Raweta’, suggesting higher concentrations of these radicals in the tolerant cultivar (Fig. 2). ZEA treatment increased the amount of radicals in the chloroplasts, and the rise was greater in the organelles obtained from the plants grown from the infected grains than from those originating from directly treated leaves. The changes were more pronounced in cv. ‘Raweta’.

### Electron paramagnetic resonance analysis

**Fig. 3:**
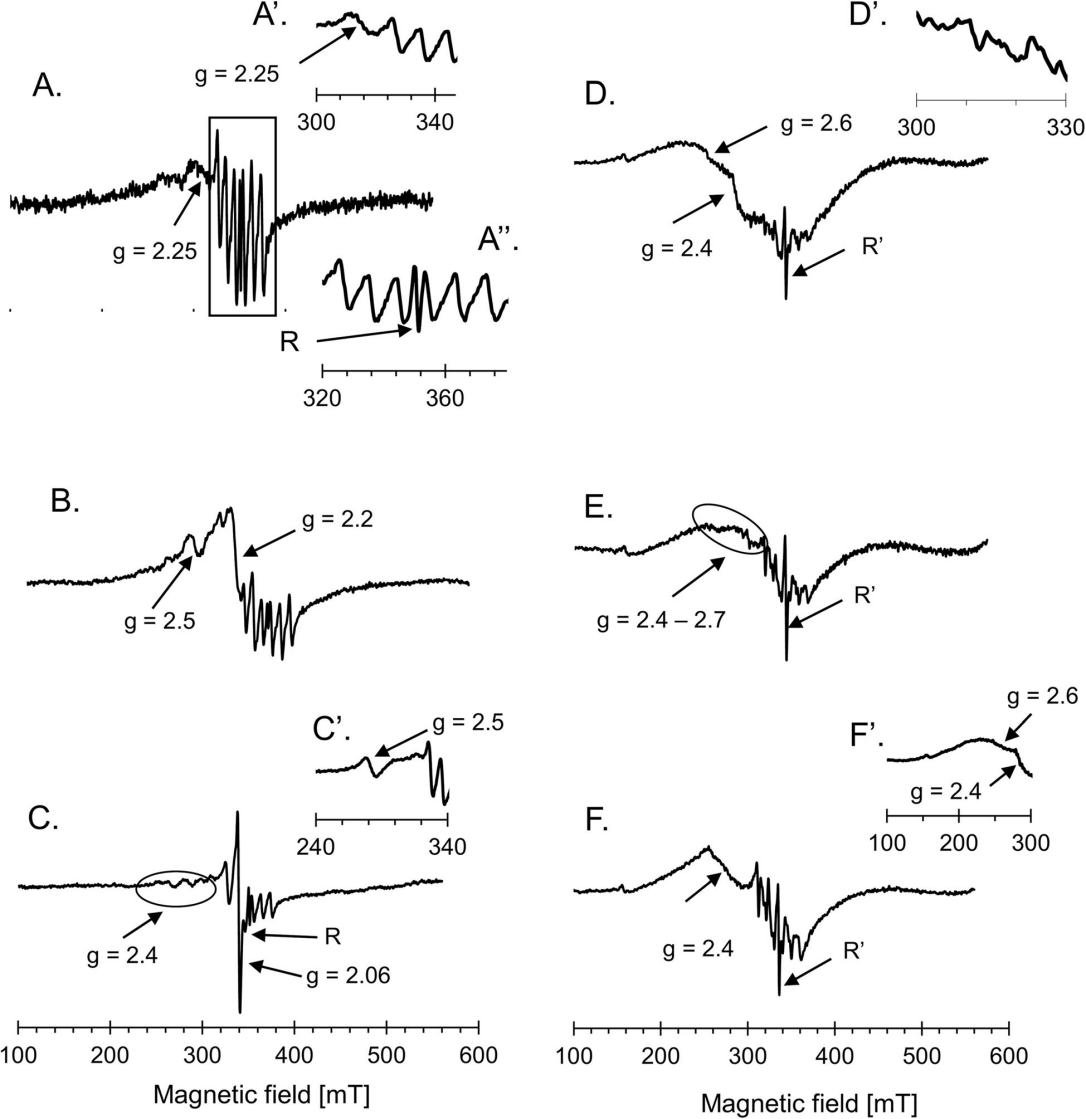
Examples of EPR spectra of cv. ‘Raweta’ leaves recorded at the range of 500 mT, measured at 293 K: A – control, B – ZEA treated grains, C – ZEA treated leaves; A’ – cv. ‘Parabola’ control (partial spectrum), A” – enlarged part of spectrum of cv. ‘Raweta’ control with HFS and R signals, C’ – infected leaves of cv. ‘Parabola’ (partial spectrum): measured at 77 K: D – control, E – ZEA treated grains, F – ZEA treated leaves; D’ – HFS structure of control cv. ‘Raweta’, F’ – infected leaves of cv. ‘Parabola’ (partial spectrum)

**Fig. 4:**
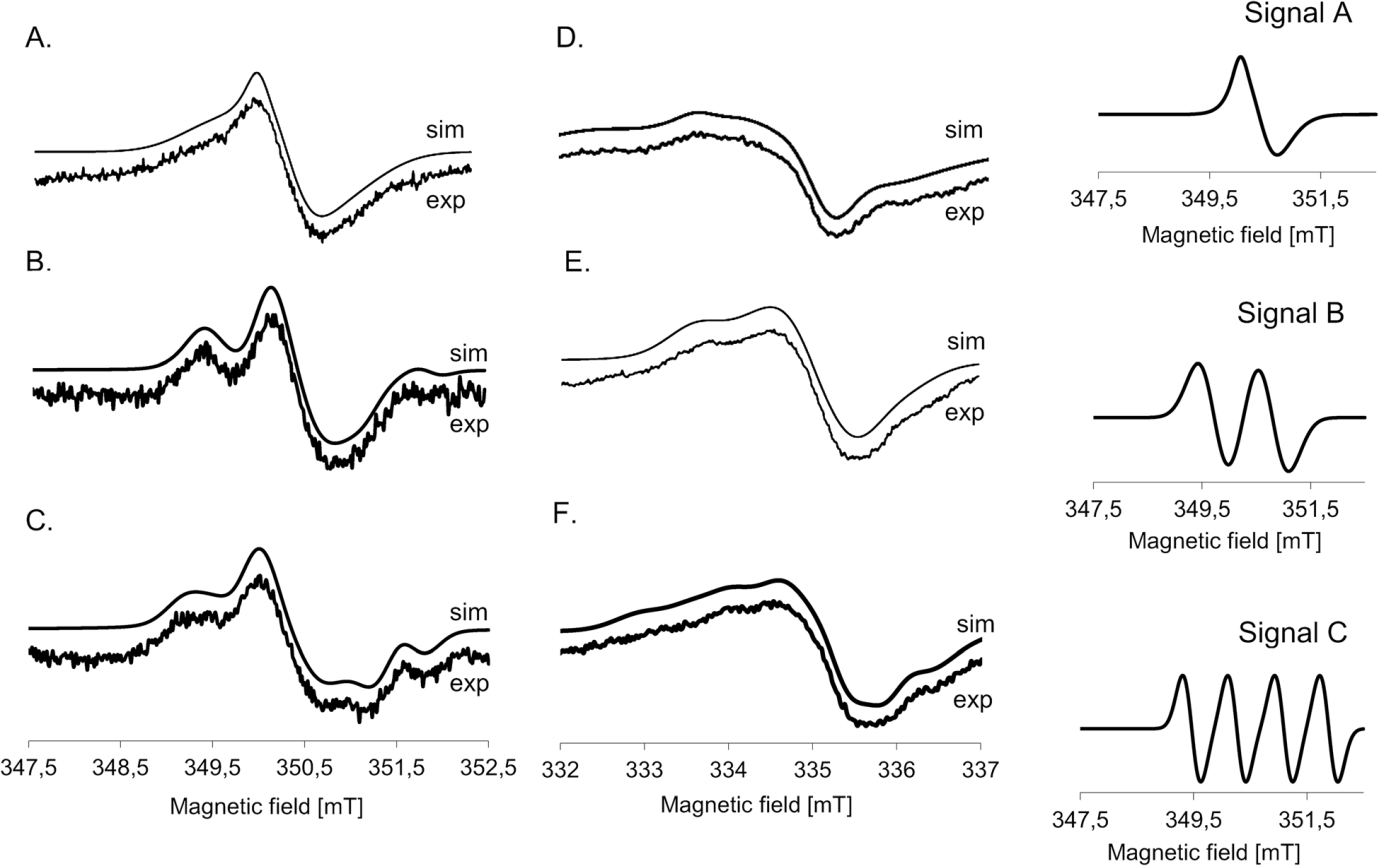
Examples of EPR spectra of organic radicals in cv. ‘Raweta’ leaves recorded at the range of 5 mT, measured at 293 K: A – control, B – ZEA treated grains, C – ZEA treated leaves; measured at 77 K: D – control, E – ZEA treated grains, F – ZEA treated leaves. Signals A, B and C were used to simulate the spectra recorded at 293 K

EPR measurements revealed that regardless of the registration temperature, the characteristic six lines of hyperfine structure (HFS), overlapping on a broader signal at g = 2.00, were observed in the spectra of control leaf samples from both wheat genotypes (Fig. 3A”, D). In the spectra recorded at room temperature, these signals were not disturbed and slightly more intense for cv. ‘Raweta’. Between the third and fourth line of the hyperfine structure, a narrow signal R at g = 2.00, with similar intensity for both genotypes, was observed. At room temperature, the line at g = 2.25 was more distinguished in the spectrum of cv. ‘Parabola’ (Fig. 3A, A’). A drop in the registration temperature to 77 K revealed broad lines at g = 2.6 and g = 2.4 that became the main signals of the spectra. Additionally, low intensity lines between the main ones of HFS became visible (Fig. 3D, D’), and a low intensity signal at g = 4.26 appeared in the spectra of both genotypes. The character of signal R changed, the line broadened and increased its intensity (signal R’) (Figs. 3D, 4D).

The leaf spectra of wheat plants grown from the grains subjected to ZEA treatment showed more significant changes for cv. ‘Raweta’. In the range of g values 2.2–2.5, new signals appeared in the spectrum recorded at room temperature (Fig. 3B). At 77 K, the signals in this range disappeared and lines at g = 2.4 and 2.6 became visible, similarly as in control samples (Fig. 3E). Simultaneously, signal R changed to R’, as in control samples.

The spectra of the leaves directly treated with ZEA were different than those of plants grown from the grains infected with this mycotoxin (Fig. 3C, F). The main change in the spectrum of cv. ‘Raweta’ recorded at room temperature involved the appearance of a strong line at g = 2.06 and four lines around g = 2.4, with small, equal intensities. In cv. ‘Parabola’ spectrum the signal at g = 2.25 vanished, and another one at g = 2.5 was noted (Fig. 3C’). In cv. ‘Raweta’ spectrum recorded at 77 K, the signal at g = 2.6 decreased and mainly the line at g = 2.4 was visible (Fig. 3F), whereas in cv. ‘Parabola’ spectrum the broad signal at g = 2.6 only slightly decreased (Fig. 3F’). In order to scrutinize the narrow line R, the spectra were recorded in the range of 5 mT.

The simulation of line R at g = 2.00 in the spectrum of control plants showed that it consisted of two components: signals A and B (Fig. 4A). Signal A, accounting for about 70% of the spectrum, exhibited small anisotropy and parameters: g_1_ = 2.0064, g_2_ = 2.0042, g_3_ = 2.0037, g_av_ = 2.0048, A_av_ = 0.2 mT, whereas signal B was isotropic, with g = 2.0050, A = 0.7 mT. The spectra of the leaves originating from the plants grown from ZEA treated grains (Fig. 4B) were composed of signals A and B with increased g values to g_av_ = 2.0054 and g = 2.0058, respectively. The anisotropy of signal A diminished, whereas the hyperfine constant for center B increased to 1.1 mT. Simultaneously, a weak signal (signal C) at g = 2.0035 split to four almost equidistant lines (A_1_ = 0.8 mT, A_2_ = 1.6 mT) with similar intensities had to be added to the simulated spectrum for its good fitting to the experimental line. Foliar ZEA treatment resulted in the spectrum (Fig. 4C) containing signal A, signal B with increasing value of HFS constant (A_1_ = 1.6 mT), and signal C with higher contribution than that found in the leaf spectrum of the plants grown from ZEA treated grains. The temperature decrease turned the signal R into a new one, significantly broader than R, marked as R’. Its parameters for control samples: g_1_ = 2.0081, g_2_ = 2.0045, g_3_ = 2.0013, g_av_ = 2.0045, A_2_^β^ = 1.7 mT (Fig. 4D) changed slightly for the signals observed in the spectra of leaves directly treated with ZEA (Figs. 4E, F).

## Discussion

Biochemical analysis of the chloroplasts isolated from the studied seedlings revealed differences in the ability of these organelles to protect plants against oxidative stress resulting from ZEA accumulation, depending on stress tolerance of wheat cultivar and the way of the mycotoxin application. In the plants grown from the seeds soaked in ZEA, the antioxidant system was less efficient, as evidenced by a decrease in the activity of the tested enzymes. Greater changes registered in cv. ‘Raweta’ confirmed its lower resistance to this stressor. Such a decrease in enzymatic activity in comparison with control may indicate partial damage to the protein structure of these enzymes [15], more pronounced in cv. ‘Raweta’. Greater resistance of cv. ‘Parabola’ may be also indicated by the increase in SOD and CAT activity after direct application of ZEA to the leaf surface. This suggests stimulation of the mechanisms protecting the plants against the formation of oxidative radicals. More abundant generation of radicals in the chloroplasts obtained from the plants grown from the seeds treated with ZEA confirmed lower efficiency of the antioxidant system during this treatment. Higher amounts of superoxide radicals observed in the chloroplasts of the sensitive cultivar might indicate lower efficiency of its antioxidant system. Interestingly, this was observed regardless of the method of ZEA application and its subsequent transport to the chloroplasts. Higher antioxidant potential of chloroplasts in the tolerant cultivar was confirmed by higher values (under control conditions) of all tested indicators (DPPH, AA, Fe (III)/Fe (II)).

DPPH appears in the presence of hydrogen donating antioxidants in the non-radical form [15]. The decrease of scavenging activity upon ZEA treatment could result from involvement of the antioxidants in the removal of ROS generated under stress. The greater decrease associated with a long-term ZEA-stress (application to grains) could be caused by a more effective synthesis of antioxidants than after short-term interaction with ZEA (foliar treatment), similarly as found during microscopic visualization of superoxides.

Ascorbic acid (AA) takes part in redox reactions in which one reactive substance is reduced at the expense of the oxidation of another [16]. Also the values of the “reducing power”, expressed as the ability to reduce Fe (III) ions to Fe (II), indicated that redox reactions in the chloroplasts under ZEA application played an important role in defense processes. The higher content of Fe (III) ions in control samples of tolerant plants was associated with the lower level of free electrons that might reduce these ions to Fe (II), and pointed to a more effective action of the antioxidant system in this cultivar. The drop in Fe (III) accumulation, caused by ZEA application (greater in the case of foliar than grain treatment), indicated the increase in free electron levels generated under the stress conditions. The decrease of reducing power was greater in the samples originating from the sensitive wheat genotype, confirming that its antioxidant system was less efficient.

Although the measurements were not performed directly in chloroplasts, the studies of EPR signals allowed for characterization of paramagnetic metal ions whose main locations and functions were associated with these organelles. Moreover, changes in redox status of metal ions upon ZEA treatment could be monitored. The analysis of EPR signals appearing in the spectra combined with literature data allowed for attribution of the observed signals to particular paramagnetic species. The signal of six hyperfine lines overlapping a broad one was ascribed to Mn species. The well resolved hyperfine structure observed at room temperature originated from freely rotating aqua - complex of Mn (II) and is often found in EPR spectra of various plants [6, 10], while the broad line was ascribed to dipole-dipole interacting Mn (II) ions situated mainly in protein matrix [17]. Signals observed in g range between 2.2 and 2.5 were attributed to inorganic antiferromagnetically coupled paramagnetic Fe (III) ions forming Fe-O-Fe clusters, ferric oxides, oxyhydroxides and/or phosphates that were accumulated in the “iron-core” of ferritin [18, 19], whereas broad signals at g = 2.4 and 2.6, appearing at 77 K, were ascribed to Fe (III) ions bonded to protein matrix in the ferritin protein shell, containing ferric and ferrous ions [20, 21]. The small line with g = 4.26, observed in all spectra recorded at 77 K, was attributed to non-hem high spin Fe (III) with rhombic symmetry [6].

**Fig. 5:**
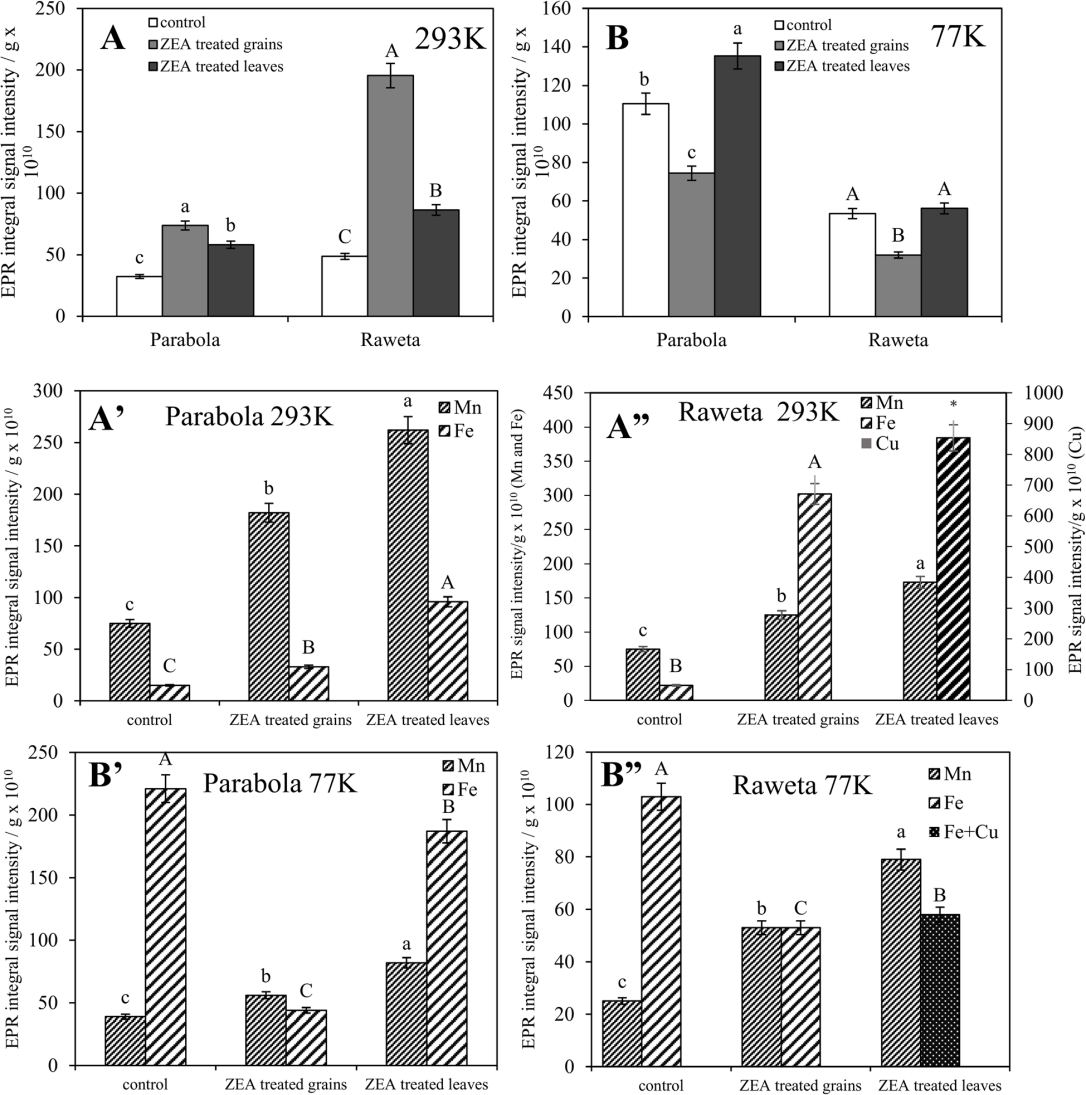
Integral signal intensity of spectra recorded in the range of 500 mT at 293 K (A) and 77 K (B) for cv. ‘Parabola’ and ‘Raweta’ leaves. The results are the means (*n* = 5) ± SE; significant differences (*P* < 0.05) between treatments separately for each genotype are marked by different letters. Intensity of Fe (III), Cu (II) and Mn (II) signals as recorded in the range of 500 mT at 293 K (A’, A”) and 77 K (B′, B”). The results are the means (*n* = 5) ± SE; significant differences (*P* < 0.05) between treatments separately for each element are marked by different letters

Calculation of intensity of particular signals, recorded at room temperature, indicated that at control conditions, the signal of Mn (II) ions significantly contributed to the spectrum, especially for cv. ‘Parabola’, in which its intensity was about four times stronger than that of Fe (III), while for cv. ‘Raweta’ it was only about 2.5 times stronger (Fig. 5A’, A”). Upon ZEA treatment the observed increase of integral spectrum intensity of cv. ‘Parabola’ leaves (Fig. 5A), measured at room temperature, was caused mainly by changes of manganese signal, whereas for cv. ‘Raweta’ it resulted from the growth of Fe (III) signals at g in 2.2–2.5 range (Figs. 3B, 5A”). The stronger increase in the signal intensity of manganese aqua complexes for cv. ‘Parabola’ than for cv. ‘Raweta’, treated with ZEA in two different manners (Fig. 5A’, A”), could suggest better accumulation of water in the tissues of the tolerant genotype. Binding of water molecules in the plant tissues may be considered one of the factors providing the anti-stress protection by ensuring the appropriate quaternary structure of proteins participating in photosynthesis. In the sensitive genotype the growing intensity of Fe (III) signal could result from oxidation of Fe (II) species by ROS generated during ZEA action (Fig. 3B, 5A’, A”). At the applied EPR measurement conditions Fe (II) ions are invisible in the spectra. The strong line at g = 2.06 and four lines around g = 2.4, situated in the place of Fe (III) signals, observed in the spectrum of cv. ‘Raweta’ leaves directly treated with ZEA, were ascribed to Cu (II) ions in square planar complexes of proteins [6] (Fig. 3C). Hence, it could be suggested that Cu(I) species, silent in EPR, were probably present in untreated plant material and underwent oxidation to Cu (II) upon ROS presence, similarly as observed for Fe species.

Contrary to room temperature, in the spectra of control plants measured at 77 K, the signals of Fe (III) ions with g equal to 2.4 and 2.6 were dominant (Fig. 5B″) and two times higher in the spectra of cv. ‘Parabola’. Upon ZEA treatment the intensity of these signals decreased, whereas that of Mn (II) signals increased for both cultivars. The changes in Fe (III) signals were more visible for cv. ‘Parabola’ when the grains were treated with ZEA, whereas for cv. ‘Raweta’ these changes were smaller. In the latter cultivar, Fe (III) signal (g = 2.4) in the spectrum of ZEA treated leaves overlapped Cu (II) signal (Fig. 3F, 5B”), hence the intensity of the line was a sum of those originating from Fe and Cu species.

Lowered amount of Fe (III) species upon ZEA treatment (Fig. 5B’, B″) resulted probably from the degradation of Fe (III) - protein complexes and was observed in all plants treated with ZEA, regardless of the way of its introduction (directly or indirectly), which confirmed that ZEA exerted a negative influence on plants. The amount of Fe (III) ions present in plants subjected to ZEA treatment was the result of two processes: degradation of ferric protein complexes (this process was visible in the spectra recorded at 77 K) and oxidation of ferrous ions probably by ROS generated upon ZEA stress (observed in the spectra measured at 293 K). The observed phenomenon indicated that the way of ZEA application influenced the mechanism of plant structure damage.

The changes of signals originating from radical species can provide further data on the influence of ZEA on wheat plants. Both overlapping signals, A and B, giving R line recorded at room temperature in the control spectra, were characteristic for carbon centered radicals located in carbohydrate molecules [5, 21]. The changes in intensity of these signals upon ZEA treatment were connected with modification of the R signal by the appearance of additional lines situated symmetrically around line R (Fig. 4B, C), which were more intense in the spectra of the leaves treated directly with ZEA. The lower value of g factor of signal C could indicate that when ZEA interacted directly with the leaves, the radicals were formed at carbohydrate molecules with lower molecular weight, whereas the species formed in the plants grown from the infected grains, giving signals with higher g factor, were situated at carbohydrates of higher molecular weight, for example starch.

**Fig. 6:**
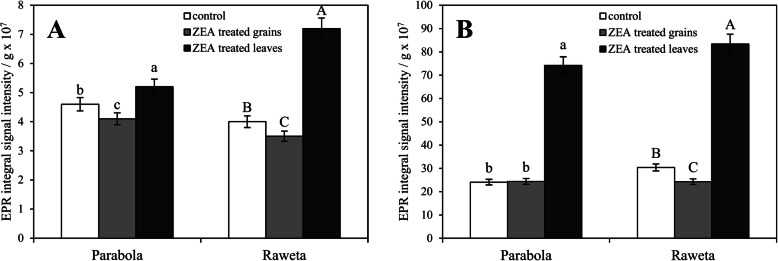
Integral signal intensity of organic radicals recorded in the range of 5 mT at 293 K (A) and 77 K (B). The results are the means (n = 5) ± SE; significant differences (*P <* 0.05) between treatments separately for each genotype are marked by different letters

Intensity of the signal R measured at room temperature was slightly lower for cv. ‘Raweta’ control plants than for cv. ‘Parabola’ ones. ZEA treatment of grains decreased it, whereas ZEA application to the leaves increased its value, more visibly in cv. ‘Raweta’ (Fig. 6A). The formation of stable carbohydrate radicals occurred as a result of transfer of free electrons from ROS, formed upon ZEA action, to carbohydrate molecules. Such a stabilization of electrons, more effective in cv. ‘Raweta’, was one of the defense mechanisms of plant against stress, as postulated by our earlier hypothesis assuming that a short direct stress increased the amount of stable carbohydrate radicals mainly in sensitive genotypes, whereas in tolerant ones other mechanisms were activated [5].

A new signal R’, recorded at 77 K (Fig. 4D-F) and observed in many plant systems, was ascribed to a stable tyrosyl radical [22], acting as an electron transfer step in biochemical redox processes [23]. Its amount increased considerably in the leaves experiencing a direct contact with ZEA, simultaneously with increasing g_1_ parameter to 2.0091, which suggested disturbance of tyrosyl radical geometry upon ZEA treatment. It was probably caused by the removal of the hydrogen atom from the hydroxyl group of the tyrosine molecule [19, 22] by reactive oxygen species formed upon ZEA stress. This suggested the destruction of organic matrices, as indicated by a disorder in biochemical surroundings of metal species, more noticeable in the sensitive genotype. The lack of significant alterations in tyrosyl radical concentration in the plants grown from the grains treated with ZEA was in line with the small changes in the amount of carbohydrate radicals, and could indicate that if more time elapsed from the moment of contact with ZEA, the redox equilibrium in plant tissues was re-established, whereas the direct stress evoked the radical transformation.

## Conclusions

EPR studies indicated that treating the plants with ZEA led to the damage of iron - protein complexes and to changes in the redox status of metal ions being important components of the antioxidant system. Moreover, we found that the mechanism of ZEA interaction with biochemical structures depended on the way of ZEA application. In the case of directly treated leaves, the defense mechanism based on the generation of stable organic radicals was initiated, mainly in the sensitive genotype, whereas plants growing from the grains treated with ZEA adapted to the disturbed tissue equilibrium probably by increasing accumulation of water and starch. These observations could suggest that the mode of defense mechanisms activated in plants depended on the method of ZEA application.

## Methods

### Chemicals

All reagents (including zearalenone) were purchased from Sigma-Aldrich Company (Germany, Munich).

### Plant material

Spring wheat of two cultivars: ‘Parabola’ and ‘Raweta’ were obtained from the Polish Plant Breeding Stations (Radzików and Strzelce, Poland). The spring wheat cultivars differed in their tolerance to oxidative stress (confirmed in our earlier experiments [10]), i.e. cv. ‘Parabola’ was the tolerant, while cv. ‘Raweta’ was the sensitive one. Also, in relation to ZEA treatment, Raweta plants characterized greater drop in fresh weight than Parabola [13]. The grains of both cultivars were germinated for 2 days in distilled water and in ZEA solution (30 μmol^.^dm^− 3^) at 20 °C, in darkness. Next, the seedlings were grown in perlite to obtain 3-leaf plants (with second well developed leaf) in greenhouse conditions, at 20/17 °C (day/night), 16 h photoperiod and 800 μmol^.^dm^− 3^ (photon) m^-2.^s^− 1^ light. Then, the pool of plants not treated with ZEA was divided into two groups. One of them was used as controls (0), and the other was the object of the experiment, in which the second leaves were covered with ZEA solution (10 μmol^.^dm^− 3^) by brushing. The measurements were performed 24 h after treatment. ZEA concentration used for the leaves was lower than that for the grain treatment, and it was chosen on the basis of Kornaś et al. [13] studies, which showed no significant differences in the structure of chloroplasts obtained from the samples exposed to intra- (grains) and extracellular (leaves) ZEA treatment. Each treatment included 10 pots with 12 plants in three independent replications. All experiments were performed on the second leaves of the seedlings.

### Chloroplast isolation

Fresh leaves collected from the plants were immediately homogenized in a buffer solution containing 50 mmol^.^dm^− 3^ Tris-HCl, 5 mmol^.^dm^− 3^ EDTA, and 0.33 mol^.^dm^− 3^ sorbitol (CIB), pre-filtered on a nylon mesh with a mesh size of 1000 μm, and centrifuged (400 x g). The supernatant was than centrifuged at 1000 x g. The chloroplast containing sludge was suspended in CIB and purified in Percol (40%/80%) gradient to obtain pure organelles, according to the procedure described earlier [24]. The entire experiment was carried at 4 °C. Visualization of superoxide radicals was performed on freshly prepared chloroplasts. For biochemical analyses the chloroplasts were frozen in liquid nitrogen and stored at − 80 °C in darkness.

### Determination of antioxidant activity

Enzymes activity (superoxide dismutases, SOD; catalase, CAT; and peroxidases, POX) were analysed according to the methods described in detail by Kornaś et al. [13]. All enzymes activity were tested spectrophotometrically (UV-Vis Evolution 220, Thermo Scientific, Waltham, USA) using KINLAB software to determine the reaction kinetics.

The chloroplasts were homogenized in methanol and centrifuged for 10 min at 1000 x g. The supernatant was used for further research. Deactivation of 2,2-diphenyl-1-picrylhydrazyl (DPPH) was analyzed spectrophotometrically (Thermo Scientific Evolution 200) at λ = 517 nm, after 30 min incubation of the extract with a mixture of 3 ml of 0.004% DPPH solution, in darkness [25].

The antioxidant activity was determined as described by Prieto et al. [26], and was based on the reduction of Mo (VI) to Mo(V) and formation of a green phosphate/Mo(V) complex at acid pH. The plant extract was incubated (95 °C for 90 min) with a solution containing 300 mol^.^dm^− 3^ sulfuric acid, 28 mmol^.^dm^− 3^ sodium phosphate and 4 mmol^.^dm^− 3^ ammonium molybdate and measured spectrophotometrically at λ = 695 nm. The antioxidant activity was calculated as mg of ascorbic acid (AA) per 1 g of FW.

Analysis of reducing power with Fe (III).

This parameter was determined in the samples containing 100 μl of the extract mixed with 2.5 ml of phosphate buffer (pH 6.6) and 2.5 ml of 1% potassium ferrocyanide, incubated at 50 °C for 20 min and centrifuged at 1000 x g for 10 min. Then, 0.1% FeCl_3_ was added to the supernatant, and centrifuged again. The samples were measured spectrophotometrically at λ = 700 nm.

### Cellular superoxide radical assay

Superoxide radicals were detected using a Cellular Superoxide Detection Assay kit (Abcam, Cambridge, UK). The isolated chloroplasts were incubated with superoxide detection mix for 30 min at 37 °C. The positive control was incubated with 0.2 ml/l pyocyanine. After washing, superoxide-positive chloroplasts (red) were observed using a Canon EOS 60D camera (Tokyo, Japan) and Delta IB-10 optical microscope set (Poland) with filter fluorescence set (× 400 magnification).

### Electron paramagnetic resonance (EPR) spectroscopy

EPR signals in the freshly collected leaves (sample weight: 0.02–0.03 g) were recorded with X-band Bruker Elexsys 500 spectrometer (Karlsruhe, Germany) with 100 kHz field modulation. The analyses were made at 293 K and 77 K, with the microwave power of 3 mW and 10 mW in the range of 5 mT and 500 mT, respectively. DPPH (2,2-diphenyl-1-picrylhydrazyl) was used as a g-factor standard. The parameter g and constants of hyperfine splitting of particular radical signals were found by the simulation procedures with SIM 32 software [27]. Integral intensities of the entire spectra and radicals, as well as intensities of signals of particular paramagnetic metal ions were determined. The accuracy of g-factor was ±0.0005 and ± 0.1 mT of A parameter for radical signals and of ±0.05 and ± 0.5 mT for g and A parameters respectively, for signals of transition metal ions.

### Statistical analysis

All data are presented as means ± SE and the significance of differences (at *P* < 0.05) was calculated from Duncan’s multiple range test and Student’s t-test using SAS, version 10.0 (SAS/STAT software).

## Data Availability

The data that support the findings of this study are included in this published article.

## Abbreviations

AA: Ascorbic acid
HFS: Hyperfine structure
CIB: Chloroplast isolation buffer
Cu: Copper
DPPH: 2,2-diphenyl-1-picrylhydrazyl
EPR: Electron paramagnetic resonance
Fe: Iron
MGDG: Monogalactosyldiacylglycerol
DGDG: Digalactosyldiacylglycerol
Mn: Manganese
SOD: Superoxide dismutases
ROS: Reactive oxygen species
ZEA: Zearalenone
